# Detrimental Effects of Environmental Tobacco Smoke in Relation to Asthma Severity

**DOI:** 10.1371/journal.pone.0018574

**Published:** 2011-05-04

**Authors:** Suzy A. A. Comhair, Benjamin M. Gaston, Kristin S. Ricci, Jeffrey Hammel, Raed A. Dweik, W. Gerald Teague, Deborah Meyers, Elizabeth J. Ampleford, Eugene R. Bleecker, William W. Busse, William J. Calhoun, Mario Castro, Kian Fan Chung, Douglas Curran-Everett, Elliot Israel, W. Nizar Jarjour, Wendy Moore, Stephen P. Peters, Sally Wenzel, Stanley L. Hazen, Serpil C. Erzurum, for the National Heart Lung Blood Institute Severe Asthma Research Program (SARP)

**Affiliations:** 1 Departments of Pathobiology, Cleveland Clinic, Cleveland, Ohio, United States of America; 2 Department of Pediatrics, University of Virginia, Charlottesville, Virginia, United States of America; 3 Respiratory Institute, Cleveland Clinic, Cleveland, Ohio, United States of America; 4 Pulmonary, Critical Care, Allergy and Immunologic Diseases, Wake Forest University, Winston-Salem, North Carolina, United States of America; 5 Department of Internal Medicine, University of Wisconsin, Madison, Wisconsin, United State of America; 6 Department of Internal Medicine, University of Texas Medical Branch at Galveston, Galveston, Texas, United States of America; 7 Internal Medicine/Pulmonary and Critical Care Medicine, Washington University, St Louis, Missouri, United States of America; 8 National Heart and Lung Institute, Imperial College School of Medicine, London, United Kingdom; 9 Division of Biostatistics, National Jewish Center, Denver, Colorado, United States of America; 10 Department of Medicine, Brigham and Women's Hospital, Boston, Massachusetts, United States of America; 11 Asthma Institute, University of Pittsburgh, Pittsburgh, Pennsylvania, United States of America; 12 Department of Cell Biology and Center for Cardiovascular Diagnostics and Prevention, Cleveland Clinic, Cleveland, Ohio, United States of America; National Jewish Health, United States of America

## Abstract

**Background:**

Environmental tobacco smoke (ETS) has adverse effects on the health of asthmatics, however the harmful consequences of ETS in relation to asthma severity are unknown.

**Methods:**

In a multicenter study of severe asthma, we assessed the impact of ETS exposure on morbidity, health care utilization and lung functions; and activity of systemic superoxide dismutase (SOD), a potential oxidative target of ETS that is negatively associated with asthma severity.

**Findings:**

From 2002–2006, 654 asthmatics (non-severe 366, severe 288) were enrolled, among whom 109 non-severe and 67 severe asthmatics were routinely exposed to ETS as ascertained by history and validated by urine cotinine levels. ETS-exposure was associated with lower quality of life scores; greater rescue inhaler use; lower lung function; greater bronchodilator responsiveness; and greater risk for emergency room visits, hospitalization and intensive care unit admission. ETS-exposure was associated with lower levels of serum SOD activity, particularly in asthmatic women of African heritage.

**Interpretation:**

ETS-exposure of asthmatic individuals is associated with worse lung function, higher acuity of exacerbations, more health care utilization, and greater bronchial hyperreactivity. The association of diminished systemic SOD activity to ETS exposure provides for the first time a specific oxidant mechanism by which ETS may adversely affect patients with asthma.

## Introduction

Asthma is a chronic inflammatory disorder of the airways involving complex interactions of cells and mediators, which lead to oxidative and nitrosative modifications of airway proteins [1], [2], [3], [4], [5] that are relevant to the initiation and maintenance of inflammation [3], [4], [5], [6], [7]. The protein modifications occur not only as a result of endogenous inflammatory processes in asthma but also from inhalation of exogenous environmental oxidants. Environmental tobacco smoke (ETS), a mixture of gases and particles from the burning cigarette and exhaled mainstream smoke [8], contains more than 10^14^ oxidative molecules per puff of smoke, including both nitric oxide and superoxide [9], [10]. The importance of ETS in the etiology of asthma in children has been established. Parental smoking is associated with poorer lung functions in asthmatic children, and the relative risk of asthma is greater in children exposed to cigarette smoking by both parents compared with smoking of neither parent [11], [12], [13], [14], [15]. Yet the evidence regarding effects of ETS on asthmatic adults is sparse, despite the fact that many asthmatic adults report ETS as a major exacerbating factor [14], [16], [17], [18], [19].

Epidemiologic studies suggest that ETS leads to greater severity of adult asthma [20], [21], [22], [23], [24], [25], [26], [27]. Experimental studies on the acute effect of ETS in adult asthma supports its contribution to exacerbation of asthma [28], [29]. However, effects in relation to asthma severity are unknown; and the mechanisms causing decline in ETS-exposed asthmatics are unclear. Evidence suggests that oxidant excess and diminished antioxidant capacity in the airway are closely associated with defining features of asthma including airflow limitation and hyper-reactivity [3], [6], [30], [31], [32]. Worsening airflow obstruction and airway remodeling events are closely associated with loss of antioxidant capacity of the superoxide dismutases (SOD; EC 1.15.1.11), which catalyze the reaction of superoxide to hydrogen peroxide [6], [30], [31], [32]. The mechanisms for loss of activity appear to be related to oxidative protein modifications, including the nitration and chlorination of tyrosine residues in the MnSOD [6], [7], [31], [33], [34], [35], [36]. Here, we hypothesized that ETS has quantifiable adverse effects on the health of well-established asthmatic individuals. In addition to quantifying the impact of ETS on morbidity, health care utilization and lung function, we also investigated potential mechanisms of effect by assessing systemic SOD activity in relation to ETS exposure.

## Methods

The study was conducted as part of the multicenter study through the NHLBI Severe Asthma Research program (SARP). The study includes 654 participants enrolled at 10 sites between 2002–2006. The protocol was approved by the Cleveland Clinic Institutional Review Board, and all procedures were monitored by an independent Data Safety Monitoring Board. All participants gave written informed consent.

### Participant Selection in SARP

Asthma severity (non-severe and severe asthma) was categorized as defined by the proceedings of the American Thoracic Society Workshop on Refractory Asthma [37]. Current smokers and former smokers with greater then 5 pack-year history were excluded.

### Study Visits

Participants were interviewed by SARP administrative staff at each clinical site [Wake Forest University, National Jewish Medical and Research Center, Imperial College, London (U.K), University of Wisconsin, Cleveland Clinic, Washington University St Louis, Emory University, Brigham & Women's Hospital, University of Pittsburgh, and University of Virginia]. The questionnaires included demographics, medical co-morbidities, smoking, medical and medication history [38]. Asthma quality of life, asthma exacerbations and health care utilization were assessed by using the Asthma Control Questionnaire (ACQ) developed by Juniper, *et al* [39]. Exposure to ETS was based on the question: “*Are you exposed to second hand smoke*?”

Pulmonary function testing was performed on an automated spirometer consistent with American Thoracic Society standards with National Health and Nutrition Examination Survey (NHANES III) reference. Subjects with a FEV_1_<55% predicted were excluded from methacholine testing due to safety concerns.

Blood was collected in a serum separation tube and centrifuged at 1500 rpm for 10 minutes. Serum was aliquoted and stored at −80 C until used in analysis.

### Antioxidant measurements

SOD activity was determined by the rate of reduction of ferri-cytochrome c, with one unit (U) of SOD activity defined as the amount of SOD required to inhibit the rate of cytochrome c reduction by 50% in the presence of a superoxide-generating system [6]. Serum cotinine levels are proportionate to the amount of tobacco smoke exposure, including secondary smoke inhalation. In this network study, not all subjects' serum was available for study. Due to limitation of serum, we were not able to measure SOD activity in all asthmatic subjects. Non -severe and severe asthmatics are proportional in number among all subjects enrolled in the study and within the subgroups with SOD measures. Importantly, there was not a selective loss of a specific population within any grouping. Glutathione peroxidase activity (GPx), total glutathione (GSH+GSSG) and eGPx protein were determined as previously described [40]. Serum cotinine was measured by high-performance liquid chromatography atmospheric pressure chemical ionization tandem mass spectrometry as previously described [41].

### Statistical Analysis

Patient characteristics, lung function, quality of life scores, medication use, and SOD measures were summarized within the subsets of control subjects and asthmatic subsets for those participants with and without ETS. Normally distributed quantitative variables were summarized using means and standard deviations, non-normally distributed variables as median (interquartile ranges), and categorical variables were summarized by their observed frequencies and percents within the participant subsets. Participants with and without ETS exposure within the subsets were compared with respect to quantitative and categorical variables using T-tests and Chi-square tests, respectively. Wilcoxon rank sum tests were a non-parametric alternative for comparisons with respect to quantitative or ordinal variables, and Fisher's exact test was an alternative to the Chi-square test in the presence of low expected frequencies in the chi-square calculations.

Logistic regression models were used to calculate odds ratios (ORs) of abnormal lung function (FEV_1_/FVC below 70, FEV_1_below 80%, change in FEV_1_ above 12% and methacholine PC_20_ below 3.1 mg/ml), morbidity and medication, with respect to ETS. The logistic models, and the two-group comparison T-test models, were extended to include the interaction between asthma severity and ETS to determine if ETS associations should be described within severe and non-severe asthmatics separately.

Multivariable regression models were constructed to study the relationships between variables and ETS and potential confounders. Age, body mass index, sex, and race (African heritage vs. non-African heritage) were included in all models as covariates. For a given quantitative or ordinal study variable chosen as the dependent variable, interactions were considered among the following variables: asthma category, ETS, sex, and race. Sequentially, likelihood ratio tests for the presence of a 4-way interaction, then the set of 3-way interactions, then the set of 2-way interactions were performed. If none of these sets of interactions were deemed significant, at a level of 0.10, then a model without interactions was considered the final model. In the presence of interactions up to a certain degree, specific interactions of that degree were then inspected, and non-significant specific interactions at the 0.10 levels were removed together to produce a final model. Associations between ETS and dependent variables were estimated from the models with 95% confidence intervals. Analyses were conducted with JMP IN 5.1 (SAS System, SAS Institute, Cary, NC) and R version 2.4.1. (R Foundation for Statistical Computing, Vienna, Austria).

## Results

### Participants

The study participants included 366 non-severe asthmatics and 288 severe asthmatics. The age range distribution was as follow: 95 children (<20 yrs), 316 young adults (20–40 yrs), 232 age (40–65 yrs,) and 11 elderly (>65 yrs). None were current smokers but 102 were former smokers with an average of years 15±1 since they last smoked and less than 2.4±0.4 pack/years. All analysis is shown for the entire population. Analyses were also performed in asthmatics excluding former smokers with similar findings. The age range was between 6 and 79 years. Participants classified as severe asthmatics were older and shorter as compared to non-severe asthmatics (TABLE 1). A substantial portion of the non-severe asthmatic group exposed to ETS was of African heritage (41.3%) as compared to the ETS unexposed group (23.4%) (TABLE 1). Other participant characteristics are shown in TABLE 1.

**Table 1:** Baseline Characteristics of Study Participants.

Characteristic	Non-severe asthma		Severe asthma	
	ETS (N = 109)	Non-ETS (N = 257)	ETS (N = 67)	Non-ETS (N = 221)
Age (yrs)	31.6±1.2	32.0±0.8	37.9±1.7	39.5±1.1
Male Sex–No. (%)	44 (40)	81 (31)	21 (31)	89 (40)
Caucasian-No. (%)	55 (50.5)	182 (70.8)*	37 (55.2)	141 (63.8)
African-No. (%)	45 (41.3)	60 (23.4)*	24 (35.8)	61 (27.6)
Asian-No. (%)	2 (1.8)	3 (1.2)	1 (1.5)	7 (3.2)
Multiple Race-No. (%)	4 (3.7)	7 (2.7)	1 (1.5)	1 (0.5)
Others-No. (%)	3 (2.7)	4 (1.5)	4 (6.0)	10 (4.5)
Unknown-No. (%)		1 (0.4)		1 (0.5)
Weight (kg)	80.5±2.5	79.4±1.5	84.1±2.8	82.5±1.8
Height (cm)	167.1±1.1	167.3±0.7	165.1±1.5	165.0±0.8
Body Mass Index	28.6±0.7	28.3±8.1	30.7±0.9	29.9±0.6
Family History of Allergic disease				
Asthma-No. (%)	72 (66.0)	156 (60.7)	46 (68.7)	125 (56.7)
Hay fever/Allergies-No. (%)	85 (78.0)	205 (80.0)	46 (68.7)	161 (72.9)
Eczema-No. (%)	30 (27.5)	79 (30.7)	21 (31.4)	74 (33.5)
Pets-No. (%)	61 (55.0)	183 (71.2)*	37 (55.2)	104 (47.1)
High Dose Corticosteroid-No. (%)	55 (50.5)	156 (60.7)	67 (100)	221 (100)
Atopy Pos-No. (%)	4.0±0.3	3.6±0.2	2.7±0.3	2.9±0.2
IgE	343.5±60.0	225.1±19.1	472.4±143.1	402.0±71.7
Total Cells - 10^6^	6.7±0.2	6.6±0.1	7.7±0.4	7.9±0.2
Neutrophils (%)	55.6±1.1	58.4±2.2	60.1±2.0	61.0±1.0
Lymphocytes (%)	32.6±1.0	34.2±1.7	28.7±1.5	27.6±0.8
Monocytes (%)	6.8±0.2	6.8±0.3	6.1±0.4	6.6±0.4
Eosinophils (%)	4.4±0.3	4.0±0.2	4.5±0.8	4.1±0.3
Basophils (%)	0.5±0.05	0.5±0.03	0.4±0.06	0.4±0.04

*Significant difference in Caucasian and African race among ETS and non-ETS group in Non-severe asthma. The P-value (P<0.05) is calculated by the Pearson chi-square test. High dose corticosteroid defined as twice a week inhaled corticosteroids and/or weekly systemic corticosteroids. “Unkown” does not wish to declare or does not know his/her ethnicity.

### Determination of Environmental Tobacco Smoke

A total of 176 asthmatic participants (26.8%) answered affirmatively to the question, “*Are you exposed to second hand smoke?*”; with regard to asthmatic classification, 109 non-severe asthmatics (30%) and 67 severe asthmatics (23%) answered affirmatively. Of the 71 asthmatic children (ages from 6–18), 13 children were exposed to ETS. When asked if participants experienced asthma symptoms when exposed to ETS, 72% of the 654 asthmatic participants reported asthma symptoms as a result of being exposed to ETS. ETS exposed participants had cotinine levels that are considered low to moderate passive smoking (median of Cotinine (ng/ml): 0.18, IQR, 0.056–0.69). Cotinine was not detectable in asthmatics whom reported no-exposure to ETS.

### Impact of ETS exposure on asthma

#### Quality of life

The Juniper questionnaire data suggest Asthmatic participants exposed to ETS had a lower total quality of life score than non-ETS-exposed asthmatics. Perhaps predictably, the greatest decrease is observed in the environmental and symptom domains. The association of ETS exposure and worse symptom scores was most pronounced in non-severe asthmatics, which are not on regular corticosteroids, which suggests that corticosteroids may influence the perception of ETS effect (TABLE 2).

**Table 2:** Quality of Life.

	Non-Severe Asthma				Severe Asthma			
	Non-ETS (N = 236)	ETS (N = 105)	Difference (95% C. I.)	P Value	Non-ETS (N = 195)	ETS (N = 63)	Difference (95% C. I.)	P Value
Total Quality of Score								
High Dose Corticosteroid: yes	4.9 (0.1)	4.7 (0.2)	−0.2 (−0.6 to 0.2)	0.35	4.1 (0.1)	3.7 (0.2)	−0.4 (−0.72 to −0.01)	0.04
High Dose Corticosteroid: no	5.3 (0.1)	4.6 (0.2)	−0.6 (−1.0 to −0.2)	0.002				
Component Score								
Activity	5.0 (0.08)	4.7 (0.12)	−0.3 (−0.6 to −0.04)	0.02	4.0 (0.1)	3.9 (0.2)	−0.2 (−0.5 to 0.2)	0.36
Emotional	5.1 (0.1)	4.7 (0.16)	−0.4 (−0.8 to −0.02)	0.04	3.9 (0.1)	3.6 (0.2)	−0.3 (−0.8 to 0.1)	0.17
Environmental	4.9 (0.1)	4.4 (0.10)	−0.5 (−0.8 to −0.2)	0.003	4.2 (0.1)	3.5 (0.2)	−0.7 (−1.14 to −0.29)	0.001
Symptom								
High Dose Corticosteroid: yes	4.9 (0.1)	4.8 (0.2)	−0.1 (−0.5 to 0.3)	0.63	4.0 (0.1)	3.6 (0.1)	−0.4 (−0.8 to −0.03)	0.03
Dose Corticosteroid: no	5.3 (0.1)	4.5 (.0.2)	−0.7 (−0.3 to −1.2)	0.001				

Values are means (SE). High dose corticosteroid defined as >twice a week inhaled corticosteroids and/or weekly systemic corticosteroids. All severe asthmatics were on high dose corticosteroids by definition of severity. For non-severe asthmatics, means (SE) shown relative to dose of corticosteroid for those values that are different by corticosteroid dose (p<0.05).

#### Lung function

Non-severe asthmatics exposed to ETS had poorer lung function, as confirmed by a significantly lower %FEV_1_ and FEV_1_/FVC (FEV_1_ −4.14, 95%CI. −8.09 to −0.20; FEV_1_/FVC −0.03, 95%CI. −0.06 to −0.01). ETS was associated with greater airway reactivity in all asthmatics exposed to ETS as compared to asthmatics not exposed. Non-severe asthmatics with ETS exposure had greater airflow reactivity to bronchodilator (%ΔFEV_1_ 3.8, 95%CI. 0.97 to 6.2, p = 0.009). Severe asthmatics exposed to ETS had greater airway hyperresponsiveness to provocative challenge with methacholine (PC_20_ −2.88, 95%CI. −4.41 to −1.24, p = 0.001) than those not exposed.

Likelihood ratio tests confirmed that ETS exposure was associated with significantly lower lung function, Specifically, non-severe asthmatics with ETS-exposure had an estimated 2.1 times higher odds for worse airflow obstruction (FEV_1_/FVC<70%) than non-exposed non-severe asthmatics (OR 2.1, 95%CI. 1.3–3.5, p = 0.003). ETS-exposure resulted in 1.7 fold greater likelihood of significant airflow reactivity, as measured by the change in FEV_1_ with bronchodilator >12% (OR 1.7, 95% CI. 1.0 to 2.7, p = 0.04). Severe asthmatics exposed to ETS had an estimated 15.3 times higher likelihood for more severe airway hyperreactivity (PC 20<3.1 mg/ml) as compared to non-exposed severe asthmatics (OR 15.3, 95%CI. 3 to 282, p = 0.0002).

#### Health care utilization

ETS exposure was associated with increased health care utilization, particularly among non-severe asthmatics: in this group, it increased risk for urgent care visits, emergency room visits, overnight hospital stays, intensive care unit admissions, and assisted ventilation (TABLE 3). ETS had no influence on health care utilization among severe asthmatics, but this population was pre-defined for selection into the severe category based upon clinical criteria that include poor lung functions, greater symptoms and health care utilization. Thus, the potential adverse effects of ETS may have been more difficult to discern in this population.

**Table 3:** Hospitalizations and Medications.

	Non-Severe Asthma				Severe Asthma			
	Non-ETS (N = 252)	ETS (N = 108)	Odd Ratio (95% C. I.)	P Value	Non-ETS (N = 211)	ETS (N = 65)	Odd Ratio (95% C. I.)	P Value
ICU Admission	11 (4.4)	19 (17.6)	4.7 (2.2 to 10.5)	<0.001	95 (44.8)	24 (36.9)	0.7 (0.4 to 1.3)	0.25
Night in Hospital	69 (27.1)	40 (37.7)	1.6 (1.0 to 2.6)	0.04	163 (75.8)	55 (82.1)	1.5 (0.7 to 3.0)	0.27
Urgent Care visit due to asthma	51 (20.0)	32 (30.2)	1.8 (1.0 to 2.9)	0.03	130 (61.0)	40 (59.7)	0.9 (0.5 to 1.7)	0.84
Assisted Ventilation	5 (2.0)	11 (10.4)	5.8 (21. to 18.9)	<0.001	42 (20)	18 (26.9)	1.5 (0.8 to 2.8)	0.24
ER visit for breathing problem	31 (12.2)	26 (23.9)	2.3 (1.3 to 4.0)	0.006	103 (47.7)	33 (49.3)	1.1 (0.6 to 1.8)	0.82
Use of rescue Inhaler								
>once a month	156 (60.70)	80 (73.4)	1.8 (1.1 to 3.0)	0.01	180 (82.2)	62 (92.5)	2.6 (1.0 to 7.9)	0.03
weekly or more	108 (42.02)	59 (54.1)	1.6 (1.0 to 2.7)	0.03	161 (73.9)	56 (83.6)	1.8 (0.9 to 3.8)	0.09

All data are presented in No (%). Intensive Care Unit (ICU), Emergency Room (ER).

#### Medications

ETS exposure was associated with increased utilization of rescue medication (TABLE 3). The greater use of rescue inhaler is consistent with the greater perception of symptoms by asthmatics exposed to ETS, the greater bronchial response to β-agonist of ETS-exposed asthmatics, and the greater bronchial hyperreactivity of ETS-exposed severe asthmatics. The greater use of rescue inhaler by ETS-exposed asthmatics, and in particular the severe asthmatics that are on maximal medical therapy, indicates an adverse effect of ETS exposure on asthma control.

### Antioxidant SOD Activity and ETS exposure

**Figure 1:**
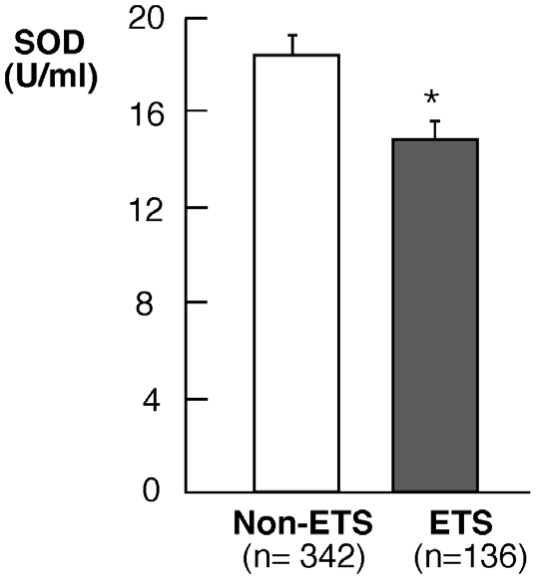
Analysis of total SOD activity in all asthmatics exposed to ETS or non-ETS. Asthmatic subjects exposed to ETS have significantly lower SOD activity (p = 0.04).

Serum SOD activity was lower in ETS-exposed asthmatic subjects than in non-exposed asthmatic subjects (SOD U/ml serum: non-ETS asthma (n = 342), 18.2±0.6; ETS asthma (n = 136), 14.7±0.9;p = 0.04) (FIGURE 1). As previously reported [6], [30], SOD concentrations were particularly low in severe asthma subjects, blunting the difference between ETS-exposure and non-ETS exposure in this group; and ETS exposure therefore had the most noticeable impact on SOD activity in non-severe asthmatics (SOD activity U/ml serum in non-severe asthma: non-ETS (n = 186), 17.7±0.9, ETS (n = 82), 14.8±1.0, p = 0.02). Of note, we have previously reported in a smaller cohort (N = 135) of this study population that asthmatics have ∼2-fold lower levels of serum SOD activity as compared to non-asthmatic healthy controls [30]. Environmental tobacco smoke exposure does not seem to alter the glutathione system (total glutathione levels, glutathione peroxidase activity or the extracellular glutathione peroxidase protein) in asthmatics. Glutathione [GSH mM: non-ETS asthma (n = 107), 1.5±0.1, ETS asthma (n = 46) 1.4±0.1; p = 0.65], glutathione peroxidase activity [GPx mU/ml: non-ETS asthma (n = 66), 0.09±0.002, ETS asthma (n = 66) 0.09±0.003; p = 0.66], and extracellular glutathione peroxidase protein [eGPx ng/ml: non-ETS asthma (n = 96) 35.9±1.5, ETS asthma (n = 44) 32.8±3.0; p = 0.30] are similar among ETS and non-ETS asthmatic subjects in this study.

### Multivariable Models and ETS

**Figure 2:**
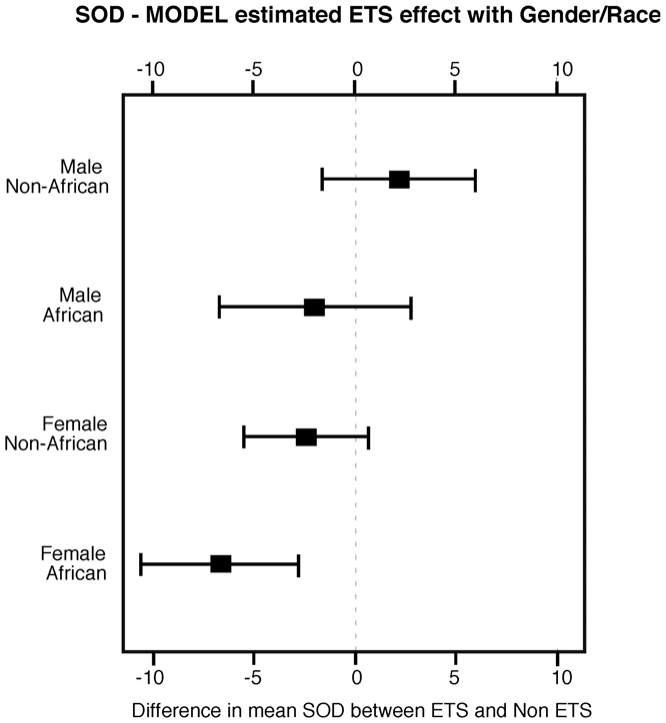
Model-estimated environmental tobacco smoke (ETS) effects on superoxide dismutase (SOD) activity. ETS exposure was associated with the lowest SOD activity for females of African heritage, which was in greatest contrast to the result for males of non-African heritage. The graph shows 95% confidence intervals for SOD activity by Gender/Race combinations.

Since age, gender, race and body mass index influence lung functions and quality of life, we applied a multivariable model to evaluate ETS effect in relation to these covariates. Despite their potential influences, these factors, including age, had no determinable impact on the associations between ETS and either quality of life or lung function. Similarly there is no association between SOD, quality of life, lung functions and age, and no detected ETS/Age interaction. On the other hand, modeling of SOD revealed a significant set of interactions, with P<0.001 for an overall test of the 6 two-way interactions formed by asthma severity, gender, race, and ETS. The implication of the interactions is that the association between ETS and SOD should be estimated within individual combinations of Gender and Race. Based on the model parameters, ETS exposure was associated with the lowest SOD activity for females of African heritage (estimated mean decrease 6.67, 95%CI. 2.73 to 10.6), which was in greatest contrast to the result for males of non-African heritage (Figure 2). The finding of greatest impact of ETS upon SOD activity in African asthmatic women suggests that the ETS-exposure may disproportionately affect this population independent of age and asthma severity.

## Discussion

This large study of a well-characterized asthma cohort provides evidence that ETS-exposure of asthmatics is associated with poorer quality of life scores, increased health care utilization and greater abnormalities in lung function. Indeed, those asthma patients traditionally characterized as non-severe were significantly closer to a severe phenotype if they were exposed to ETS. Taken together, these observations identify that ETS is an environmental factor that contributes to severity of asthma.

Etiological studies of adults have found an increased risk of asthma in relation to exposure to ETS [12], [15], [42], [43]. The Swiss Study on Air Pollution and Lung Diseases in Adults (SAPALDIA) suggests that the association of ETS and wheezing is dose-dependent [42]. Similarly, the European Community Respiratory Health Survey (ECRHS) and the Studio Epidemiologico su Ambiente e salute nelle donne (SEASD) identify associations between ETS and respiratory symptoms and/or diseases [14], [44]. A longitudinal study of Seventh-day Adventists in the United States reports an odds ratio of 1.45 for asthma in relation to 10 years of exposure to ETS in the workplace [45]. Observational studies suggest that ETS exposure is related to more severe airflow obstruction in adult asthmatics [22], [46], [47]. In addition, experimental studies identify acute effects of ETS on lung function of adults with established asthma [8], [29], [48], [49], [50], [51]. Some studies have suggested that the effect of ETS exposure may be gender related [43], [52]. Xu *et al* showed that in sex-specific regression analyses, effects of ETS appeared more pronounced in men than in women, though the interaction of gender and ETS was not statistically significantly [53]. In this study, no gender difference was found in the association between ETS and lung functions, which is in agreement with the European Community Respiratory Health Survey (ECRGH) [14].

Passive or active cigarette smoke is injurious to the lower respiratory tract by increasing the oxidant burden [31] and reducing levels of nonenzymatic antioxidants, such as vitamin C [54], and carotenoids [54], [55], [56]. Nitrosative stress characteristic of cigarette smoke can inhibit the activity of at least one SOD isoform through tyrosine nitration [57], [58], though other mechanisms may be operative as well. Consistent with our findings, Aycicek et al reported that ETS exposure leads to a decrease of total serum antioxidant capacity, which measures the total amount of radicals that can be scavenged by nonenzymatic antioxidants [59]. To our knowledge, however, this study is the first report demonstrating lower levels of specific enzymatic activity in the blood of asthmatic individuals exposed to ETS. While we do not discriminate the SOD form affected in this report, the protein levels of CuZnSOD and MnSOD among the ETS and non-ETS asthmatics were not significantly different (all p>0.05, data not shown). This is consistent with prior studies that showed loss of SOD activity in lungs of asthmatics without a decrease in protein expression, i.e. loss of activity is related to protein inactivation [6], [7], [32], [60]. The association of ETS effect on SOD activity in asthmatic women of African heritage in this study implies the possibility of a genotype-environment-phenotype interaction. In support of this concept, previous study has shown a positive association between genetic polymorphism in the MnSOD gene and the risk of cancer in cigarette smokers [61]. Furthermore, an increase in EC-SOD protein in serum of individuals with smoking-related chronic obstructive pulmonary disease (COPD) is related to polymorphisms of the EC-SOD gene [62]. Polymorphisms are also common in the CuZnSOD gene and associated with reduced affinity of the enzyme for binding Zn, which leads to greater susceptibility to oxidant damage [63]. Recent study by Arcaroli et al. showed that polymorphisms in EC-SOD are associated with acute lung injury and mortality [64]. Nevertheless, using information from a Genome Wide Association Study (GWAS) on a cohort of subjects, SOD activity had no association with SNPs within any of the three SOD isoforms within Aftrican American or Caucasian populations (data not shown). Further studies are needed to determine if individual susceptibility to ETS exposure in asthma is related to gene polymorphisms.

The American Thoracic Society guidelines define severe asthma as asthma that requires high-dose inhaled and/or systemic corticosteroid treatment and that, despite these interventions, is characterized by persistent and/or life-threatening symptoms [37]. Here, we have found that ETS exposure is associated with phenotypic features of severe asthma symptoms among those who do not formally meet criteria for severe asthma. That is to say, the adverse effects of ETS are most noticeable in non-severe subjects. We speculate that changes caused by ETS in severe asthmatics may be less evident because those patients start from a more severe baseline. However, it is also possible that severe asthmatics avoid or limit exposure to ETS due to the perceived or potential deleterious effects on their health. For example, the greater bronchial hyperresponsiveness in the ETS-exposed severe asthmatics may lead to greater sensitivity to inhalation of irritants and attempts to minimize exposure. Taken together with prior epidemiologic studies [19], [25], [27], the present study establishes that ETS significantly increases the adverse effects of asthma on individuals and the health care costs associated with managing asthmatic patients.
